# Characterization of captive and wild 13-lined ground squirrel cecal microbiotas using Illumina-based sequencing

**DOI:** 10.1186/s42523-021-00154-9

**Published:** 2022-01-03

**Authors:** Edna Chiang, Courtney L. Deblois, Hannah V. Carey, Garret Suen

**Affiliations:** 1 Microbiology Doctoral Training Program, Department of Bacteriology, University of Wisconsin-Madison, Madison, WI 53706 USA; 2 Department of Comparative Biosciences, School of Veterinary Medicine, University of Wisconsin-Madison, Madison, WI 53706 USA; 3 Present Address: Department of Bacteriology, University of Wisconsin-Madison, Madison, WI 53706 USA

**Keywords:** Hibernation, Wild, Captive, Illumina, Torpor, Microbiota, Host-microbe symbiosis

## Abstract

**Background:**

Hibernating animals experience extreme changes in diet that make them useful systems for understanding host-microbial symbioses. However, most of our current knowledge about the hibernator gut microbiota is derived from studies using captive animals. Given that there are substantial differences between captive and wild environments, conclusions drawn from studies with captive hibernators may not reflect the gut microbiota’s role in the physiology of wild animals. To address this, we used Illumina-based sequencing of the 16S rRNA gene to compare the bacterial cecal microbiotas of captive and wild 13-lined ground squirrels (TLGS) in the summer. As the first study to use Illumina-based technology to compare the microbiotas of an obligate rodent hibernator across the year, we also reported changes in captive TLGS microbiotas in summer, winter, and spring.

**Results:**

Wild TLGS microbiotas had greater richness and phylogenetic diversity with less variation in beta diversity when compared to captive microbiotas. Taxa identified as core operational taxonomic units (OTUs) and found to significantly contribute to differences in beta diversity were primarily in the families *Lachnospiraceae* and *Ruminococcaceae*. Captive TLGS microbiotas shared phyla and core OTUs across the year, but active season (summer and spring) microbiotas had different alpha and beta diversities than winter season microbiotas.

**Conclusions:**

This is the first study to compare the microbiotas of captive and wild rodent hibernators. Our findings suggest that data from captive and wild ground squirrels should be interpreted separately due to their distinct microbiotas. Additionally, as the first study to compare seasonal microbiotas of obligate rodent hibernators using Illumina-based 16S rRNA sequencing, we reported changes in captive TLGS microbiotas that are consistent with previous work. Taken together, this study provides foundational information for improving the reproducibility and experimental design of future hibernation microbiota studies.

**Supplementary Information:**

The online version contains supplementary material available at 10.1186/s42523-021-00154-9.

## Introduction

Host-microbe symbioses are dynamic, especially in animals that experience extreme shifts in diet as these changes dramatically alter substrate availability for their gut microbiota. An example includes hibernating mammals. Hibernation is an ecophysiological strategy to survive times of reduced resource availability and high-energy demand by altering both behavior and physiology. In small mammalian hibernators such as the 13-lined ground squirrel (TLGS; *Ictidomys tridecemlineatus*), the circannual hibernation cycle involves periods of summer hyperphagia when the host acquires adequate fat stores for energy usage during winter hibernation [1]. During summer, the gut microbiota has access to both dietary and host-derived substrates as rich sources of energy. In winter, the host enters hibernation, fasts for several months, and relies entirely upon fat stores for energy. The winter hibernation season is characterized by cycling between periods of depressed and normal metabolism (torpor and interbout arousal, respectively). Torpor occurs when metabolism slows to < 4% of normal rates, causing body temperature (T_b_) to plummet to just above ambient temperature (< 10 °C) [2, 3]. Torpor is interrupted by brief interbout arousals (IBAs) that can last 12–24 h [2–4]. During IBAs, metabolism increases to normal rates and T_b_ warms to ~ 36 °C [2–4]. Due to fasting during hibernation, the gut microbiota is forced to rely solely on host-derived substrates (e.g., mucins) as its source of energy. The hibernation season ends with the emergence aboveground in spring and the resumption of normal metabolic activity and feeding patterns. This natural cycle of extreme changes in diet and physiology makes mammalian hibernators like the TLGS useful systems for studying host-microbe symbioses.

The majority of our current knowledge of the hibernator gut microbiota comes primarily from studies of captive animals. Captive environments differ from natural, wild environments due to necessary changes in diet, habitat, rearing conditions, and exposure to environmental microbes [5]; as a result, the microbiotas of captive animals likely differ substantially from those of wild animals. While the use of captive animals allows for controlled experiments that have led to significant advances in our understanding of their biology, their use for gut microbiota studies reduces confidence in the conclusions that can be drawn about the microbiota’s role in the physiology of wild animals. Moreover, these limitations may restrict the ability to translate discoveries using captive host-microbial relationships to other systems, such as the use of captive mice as a model for human health [5–8]. Differences in diet are of particular concern as diet is a known major driver of microbiota composition and metabolism [9–17] and it is difficult to recapitulate a hibernator’s wild diet in a captive setting. Additionally, the development of an animal’s microbiota early in life can have long-lasting effects on microbiota composition and host physiology [13, 18, 19], and many hibernation studies use animals born in the lab rather than in the wild. Although there is a growing number of studies comparing the microbiotas of captive and wild animals [5–8, 20–55], very few have been conducted with hibernating species. One study that examined three species of hibernating bats found that the number of operational taxonomic units (OTUs) is higher in captive than in wild bats [45]. Studies in other animals have reported that differences between captive and wild microbiotas are species-specific [5, 30, 36, 53]. Therefore, it is important to understand how captivity alters the microbiota of hibernating animals like the TLGS.

Most rodent hibernation microbiota studies to date have used either 454 pyrosequencing [56–59] or clone libraries [60]. However, advances in sequencing technology have resulted in the vast majority of microbiota studies using Illumina-based sequencing technology. Because different sequencing technologies may introduce biases, it is important to compare results among sequencing methods to understand how to interpret past data in the context of newer studies. Previous comparisons of 454 pyrosequencing and Illumina sequencing data demonstrate that the two methods result in similar beta diversity results [61–63]. This is also the case for alpha diversity for samples with high diversity but not for those with low diversity [62, 63]. Results from these two sequencing platforms also produce differences in microbiota taxonomic composition, with some studies reporting differences at the genus level [62] while others reveal differences at the phylum level [63]. Therefore, it is important to consider how sequencing results from different platforms may differ in order to improve data interpretation.

Here, we used Illumina 16S rRNA gene sequencing to compare the bacterial cecal microbiotas of captive and wild TLGS in the summer. We hypothesize that wild TLGS microbiotas are more diverse than captive TLGS microbiotas, likely because the wild diet is more diverse than the captive diet. As this study is, to our knowledge, the first analysis of Illumina-based sequencing from an obligate rodent hibernator across active and hibernation seasons, we also reported changes in captive TLGS microbiotas across the year (summer, winter, and spring).

## Results

### Sequence coverage

We sequenced a total of 59 samples, which consisted of 30 cecal content and 29 cecal mucosa. Bacterial amplicon sequencing generated a total of 3,252,557 raw sequences with an average of 54,209 ± 16,411 sequences per sample (mean ± SE; range 534–972,021). Sequence clean-up in mothur resulted in a total of 2,211,103 sequences for an average of 36,852 ± 13,449 sequences per sample (range 291–796,002). After normalization, 57 samples remained (29 content, 28 mucosa) with a range of reads = 10,453–11,159. All samples had Good’s coverage ≥ 95%, which indicated sufficient sequence coverage [64].

### Cecal content and mucosa microbiotas are similar

To determine whether cecal content and mucosa microbiotas differ, we used paired tests to examine alpha and beta diversity within each of the five experimental groups. There were no differences in the number of observed OTUs, Faith’s phylogenetic diversity, phylogenetic evenness, or Shannon’s diversity (all paired t-test adj *P* ≥ 0.889, Additional file 1: Fig. S1). Similarly, we found no differences in the variances of weighted UniFrac, unweighted UniFrac, and Bray–Curtis dissimilarity (all betadisper adj *P* ≥ 0.293; Additional file 1: Fig. S2). We then examined the centroids of the three beta diversity metrics by including both squirrel ID and sample type in PERMANOVA tests. Squirrel ID accounted for the paired nature of the data and was significant for all three metrics in the captive summer group (all PERMANOVA adj *P* = 0.002) and for Bray–Curtis dissimilarity in the IBA group (adj *P* = 0.006), but not in the remaining groups and metrics (Additional file 2: Table S6). Within these significant groups and metrics, squirrel ID explained more variation in beta diversity than did sample type (Additional file 2: Table S6; all squirrel ID R^2^ ≥ 0.224, all sample type R^2^ ≤ 0.056). After accounting for squirrel ID, there were no differences in sample type for the three metrics (Additional file 2: Table S6; all adj *P* ≥ 0.912). Because sample type did not significantly impact alpha and beta diversity, we present data from mucosa microbiotas here and included those from cecal content in the supplementary materials.

### Captive and Wild microbiotas have different alpha and beta diversities

**Fig. 1:**
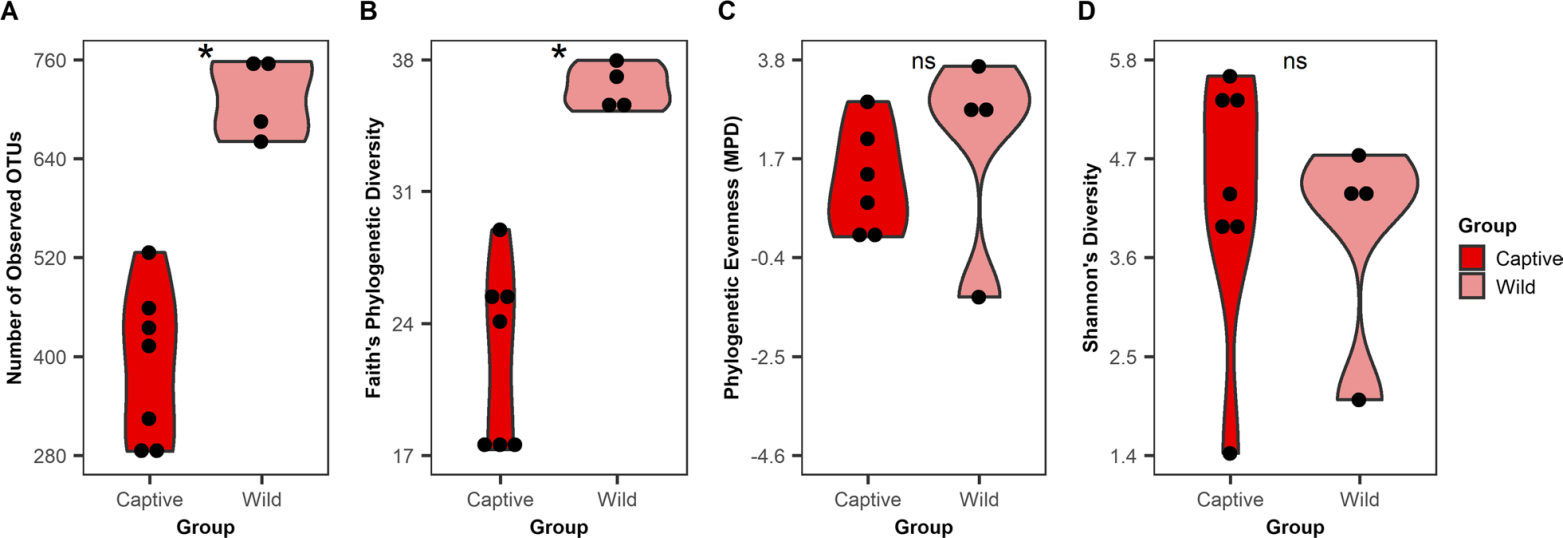
Alpha diversity of summer Captive and Wild mucosa microbiotas. Violin plots display four alpha diversity metrics: **A** the number of observed OTUs, **B** Faith’s phylogenetic diversity, **C** phylogenetic evenness (MPD), and **D** Shannon’s diversity. An asterisk indicates a significant difference (adj *P* < 0.05) and “ns” indicates no significant difference (adj *P* > 0.05). Adjusted *p*-values were calculated from t-tests followed by false discovery rate correction with the Benjamini–Hochberg procedure

We examined alpha diversity (within-sample diversity) between summer Captive and Wild groups by using four metrics (Fig. 1). In mucosa microbiotas, the number of observed OTUs was higher in Wild compared to Captive (Fig. 1A; T-test adj *P* < 0.001). Faith’s phylogenetic diversity, which is positively correlated with richness, also revealed the same difference as seen in number of observed OTUs in Wild vs. Captive (Fig. 1B; adj *P* < 0.001). There were no differences in phylogenetic evenness (Fig. 1C; adj *P* = 0.303) or Shannon’s diversity (Fig. 1D; adj *P* = 0.614). The same results were seen in content microbiotas (Additional file 1: Fig. S3).

**Fig. 2:**
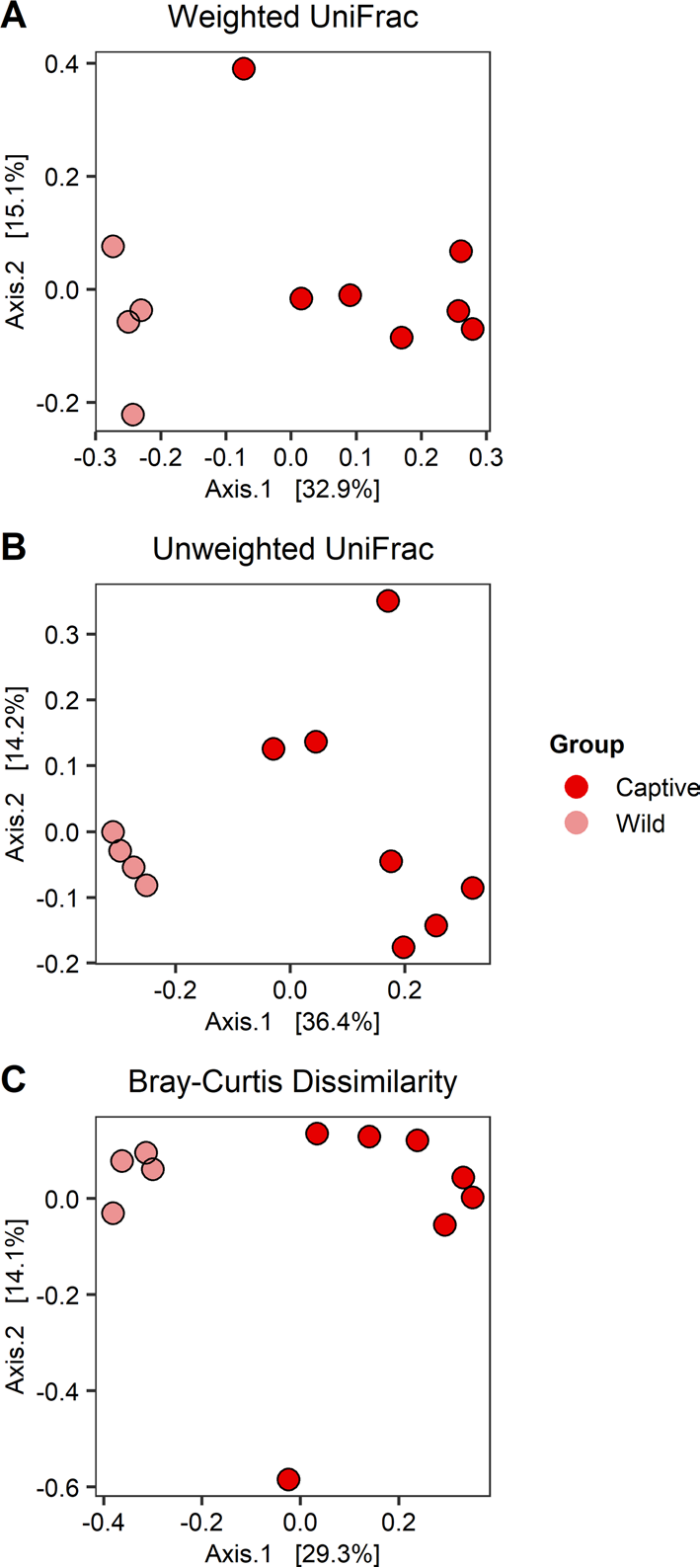
Beta diversity of summer Captive and Wild mucosa microbiotas. **A** Weighted UniFrac, **B** unweighted UniFrac, and **C** Bray–Curtis dissimilarity are displayed on PCoA ordinations. Groups are depicted with different colors

We examined beta diversity by using weighted UniFrac, unweighted UniFrac, and Bray–Curtis dissimilarity (Fig. 2). In mucosa samples, the variance of unweighted UniFrac differed between Captive and Wild groups (Fig. 2; betadisper adj *P* = 0.012), but no group differences were observed in weighted UniFrac or Bray–Curtis Dissimilarity (Fig. 2; both adj *P* = 0.241). However, the centroids in all three metrics were different (Fig. 2; all adj *P* = 0.004). Content microbiotas had different variances and centroids between Captive and Wild groups in all three metrics (Additional file 1: Fig. S4; all betadisper adj *P* = 0.006, all PERMANOVA adj *P* = 0.014).

### Few OTUs drive beta diversity differences between Captive and Wild microbiotas

To identify specific taxa that contributed to the differences between Captive and Wild microbiotas, we first examined phyla relative abundances. There were 10 phyla with total relative abundances > 1% across all mucosa samples (Table 1). Dominant phyla included the *Firmicutes* and *Bacteroidetes*, with smaller contributions from the *Cyanobacteria*, *Tenericutes*, *Verrucomicrobia*, *Proteobacteria*, *Epsilonbacteraeota*, *Actinobacteria*, *Elusimicrobia*, and *Kiritimatiellaeota*. We did not detect the *Epsilonbacteraeota* or *Kiritimatiellaeota* (Additional file 2: Table S7) in content microbiotas. Mucosa and content microbiotas had similar phyla relative abundances between Captive and Wild microbiotas (all adj *P* > 0.050); however, the phyla *Proteobacteria* and *Cyanobacteria* displayed a trend in content microbiotas (both adj *P* = 0.055).

**Table 1:** Phyla relative abundances in summer Captive and Wild mucosa microbiotas

Phylum	Relative abundance (%)		
	Captive	Wild	Adj *P*
*Firmicutes*	57.556 ± 1.923	65.567 ± 4.676	0.370
*Bacteroidetes*	36.091 ± 1.714	24.644 ± 4.707	0.295
*Cyanobacteria*	0.818 ± 0.241	3.269 ± 0.576	0.182
*Tenericutes*	0.997 ± 0.361	1.903 ± 0.659	0.433
*Verrucomicrobia*	1.425 ± 0.342	1.046 ± 0.364	0.574
*Proteobacteria*	1.359 ± 0.352	0.885 ± 0.081	0.713
*Epsilonbacteraeota*	0.124 ± 0.081	0.683 ± 0.471	0.295
*Actinobacteria*	0.175 ± 0.039	0.276 ± 0.058	0.370
*Elusimicrobia*	0.020 ± 0.006	0.293 ± 0.111	0.295
*Kiritimatiellaeota*	0.172 ± 0.167	0.014 ± 0.014	0.743
*Unclassified*	1.262 ± 0.801	1.421 ± 0.569	0.433

Phyla have total relative abundances > 1% and are displayed from overall highest relative abundance to lowest relative abundance. Relative abundances are displayed as mean ± standard error and adjusted *p*-values are from t-tests or Wilcoxon tests (depending on data normality) and adjusted for false discovery rate using the Benjamini–Hochberg procedure

As we saw no differences at the phylum-level, we proceeded at the OTU-level. Analysis of similarity percentages (SIMPER) in mucosa microbiotas identified three OTUs that contributed to differences in Bray–Curtis dissimilarity (Table 2). They explained 4.716% (cumulative SIMPER) of the difference in Bray–Curtis dissimilarity. Two of these OTUs belonged to the family *Lachnospiraceae* and one to the genus *Lactobacillus*. Both *Lachnospiraceae* OTUs had higher relative abundances in Wild compared to Captive, whereas the opposite was true for the *Lactobacillus* OTU (Table 2).

**Table 2:** OTUs that significantly contribute to Bray–Curtis dissimilarity between summer Captive and Wild mucosa microbiotas

OTU	Most resolved taxonomic classification	SIMPER percentage (%)	Captive relative abundance (%)	Wild relative abundance (%)	Adj *P*
Otu00035	*Lactobacillus*	1.881	3.170	0.074	0.033
Otu00096	*Lachnospiraceae*	1.288	0.029	2.149	0.033
Otu00119	*Lachnospiraceae*	1.547	0.000	2.547	0.026

OTUs were identified with SIMPER and statistically tested with Kruskal–Wallis tests. *P*-values were corrected for false discovery rate using the Benjamini–Hochberg procedure. Only OTUs that accounted for ≥ 1% of the differences in beta diversity and had adj *P* < 0.05 were considered significant

Content microbiotas had four OTUs that explained 7.260% of the Bray–Curtis dissimilarity (Additional file 2: Table S8). Three of these OTUs belonged to the family *Lachnospiraceae*, one of which was further classified to the genus *Lachnospiraceae NK4A136 group*, and they were not detected in Captive microbiotas (Additional file 2: Table S8). The remaining OTU was classified to the genus *Lactobacillus* and had higher relative abundance in Captive than in Wild (Additional file 2: Table S8). Two OTUs (one *Lachnospiraceae* and one *Lactobacillus*) were significant in both content and mucosa microbiotas.

### Wild microbiotas have more core OTUs than Captive microbiotas

The Wild mucosa microbiota had 323 core OTUs whereas the Captive mucosa microbiota had 43 (Additional file 2: Table S9). The majority of the Wild core OTUs were classified to the families *Ruminococcaceae* (100 OTUs), *Lachnospiraceae* (93 OTUS), *Muribaculaceae* (34 OTUs), unclassified *Gastranaerophilales* (17 OTUs), and *Rikenellaceae* (14 OTUs). The majority of the Captive core OTUs were classified to the families *Ruminococcaceae* (15 OTUs) and *Lachnospiraceae* (10 OTUs). There were 24 shared core OTUs across all mucosa samples (Additional file 2: Table S9). All but one of these were classified to the order *Clostridiales* and the most common family was the *Ruminococcaceae* with 10 OTUs. The sole non-Clostridia OTU was classified to the genus *Parasutterella*. Content microbiotas had similar numbers and taxonomic classifications of core OTUs (Additional file 2: Table S10).

### Captive microbiotas across seasons have similar phyla and core OTUs

Across all mucosa samples in the four captive groups, we identified 10 phyla with total relative abundances > 1% (Table 3). Only the *Firmicutes* differed among groups, with higher relative abundances in Summer compared to Torpor or IBA (both Tukey’s HSD adj *P* < 0.001). Phyla with no differences among groups included the *Bacteroidetes*, *Verrucomicrobia*, *Proteobacteria*, *Kiritimatiellaeota*, *Cyanobacteria*, *Tenericutes*, *Actinobacteria,* *Elusimicrobia,* and *Epsilonbacteraeota*. Content microbiotas had the same phyla, but we did not detect the *Epsilonbacteraeota* (Additional file 2: Table S11). In these samples, both the *Firmicutes* and *Bacteroidetes* differed among groups. The *Firmicutes* had higher relative abundances in Summer compared to Torpor or IBA (Additional file 2: Table S11; Dunn’s test adj *P* = 0.0012 and 0.001, respectively); the opposite was true for the *Bacteroidetes* (Additional file 2: Table S11; Tukey’s HSD Summer and Torpor adj *P* = 0.006, Summer and IBA adj *P* = 0.017). No other phyla differed among groups.

**Table 3:** Phyla relative abundances in mucosa microbiotas of captive groups

Phylum	Relative abundance (%)				Significant Comparisons
	Summer	Torpor	IBA	Spring	
*Firmicutes*	57.981 ± 1.910	30.611 ± 4.687	30.884 ± 3.045	45.113 ± 3.419	Summer-Torpor
					Summer-IBA
*Bacteroidetes*	35.592 ± 1.652	45.203 ± 5.749	41.836 ± 2.778	45.942 ± 3.062	–
*Verrucomicrobia*	1.405 ± 0.336	12.219 ± 5.786	19.152 ± 6.099	4.266 ± 2.049	–
*Proteobacteria*	1.355 ± 0.347	3.282 ± 0.245	3.467 ± 0.740	1.955 ± 0.544	–
*Kiritimatiellaeota*	0.171 ± 0.164	5.523 ± 3.395	1.358 ± 0.978	0.809 ± 0.674	–
*Cyanobacteria*	0.807 ± 0.238	1.012 ± 0.241	0.482 ± 0.192	0.365 ± 0.274	–
*Tenericutes*	1.019 ± 0.361	0.103 ± 0.036	0.153 ± 0.054	1.197 ± 0.541	–
*Actinobacteria*	0.189 ± 0.035	0.125 ± 0.049	0.228 ± 0.048	0.145 ± 0.054	–
*Elusimicrobia*	0.020 ± 0.005	0.118 ± 0.079	0.538 ± 0.281	0.000 ± 0.000	–
*Epsilonbacteraeota*	0.122 ± 0.079	0.144 ± 0.135	0.018 ± 0.018	0.000 ± 0.000	–
*Unclassified*	1.339 ± 0.800	1.657 ± 1.573	1.883 ± 1.589	0.208 ± 0.055	–

Phyla have total relative abundances > 1% and are displayed from overall highest relative abundance to lowest relative abundance. Relative abundances are displayed as mean ± standard error. Significant comparisons had adjusted *p*-values < 0.05 based on ANOVA and Tukey's HSD tests, or had adjusted *p*-values < 0.025 based on Kruskal–Wallis and Dunn's Tests, depending on data normality

In the captive mucosa microbiotas, we identified 43 core OTUs in Summer, 65 in Torpor, 60 in IBA, and 141 in Spring (Table 4). Core OTUs in each group were dominated by the families *Lachnospiraceae* and *Ruminococcaceae* (Additional file 2: Table S12). Torpor and Spring also had > 10 core OTUs classified to the family *Muribaculaceae*. There were 16 core OTUs found in every mucosa sample. The content microbiotas had similar results (Additional file 2: Table S13), and 10 core OTUs found in all content samples were also found in all mucosa samples.

**Table 4:** The number of core OTUs in mucosa microbiotas of each captive group and shared between groups

	Summer	Torpor	IBA	Spring
Summer	43	24	26	34
Torpor		65	35	49
IBA			60	41
Spring				141

### Captive microbiotas in active and winter groups have different alpha and beta-diversities

**Fig. 3:**
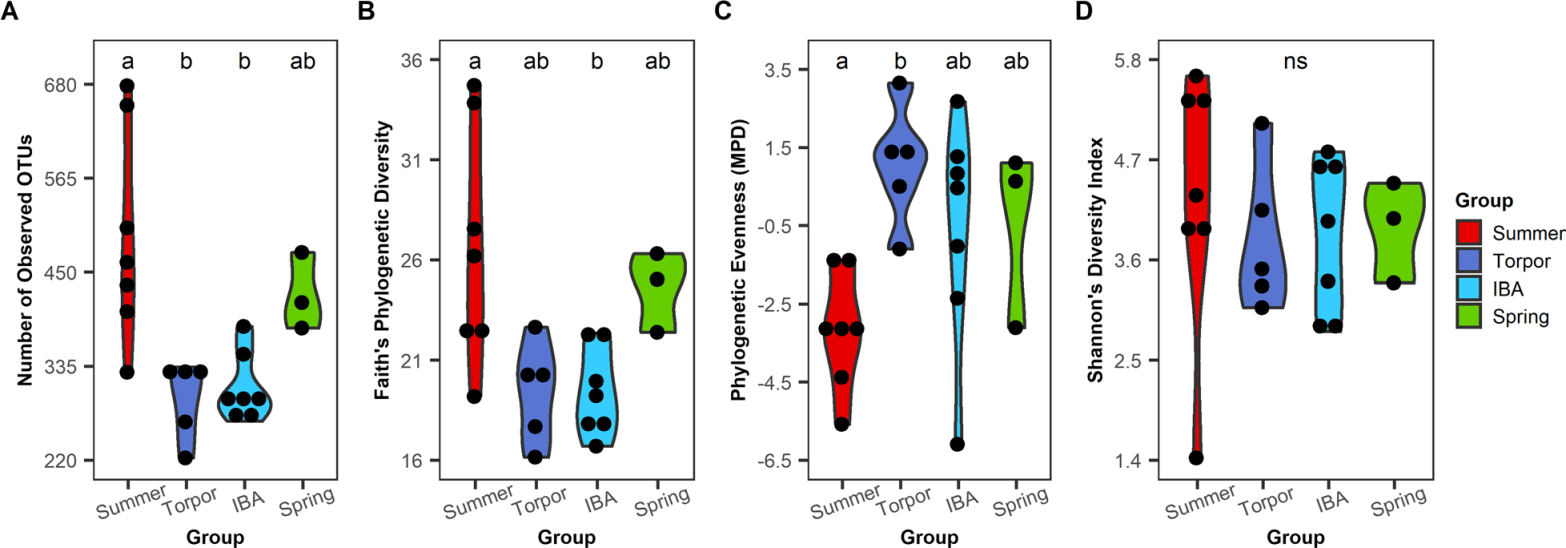
Alpha diversity comparison among mucosa microbiotas of captive groups. Violin plots display four alpha diversity metrics: **A** the number of observed OTUs, **B** Faith’s phylogenetic diversity, **C** phylogenetic evenness (MPD), and **D** Shannon’s diversity. Groups that share a letter are not significantly different (adj *P* > 0.05), whereas groups that share no letters are significant different (adj *P* < 0.05). Metrics with no significant comparisons among groups are indicated with “ns.” Adjusted *p*-values were calculated from ANOVA and Tukey’s HSD tests, or from Kruskal–Wallis and Dunn’s tests, depending on data normality

Summer mucosa microbiotas had more observed OTUs (Fig. 3A) compared to Torpor or IBA (Dunn’s test adj *P* = 0.006 and 0.010, respectively), but did not differ from Spring (adj *P* = 0.450). Torpor was not different from IBA or Spring (adj *P* = 0.408 and 0.043, respectively), and IBA was not different from Spring (adj *P* = 0.039). Phylogenetic diversity (Fig. 3B) was higher in Summer than IBA (adj *P* = 0.018), whereas phylogenetic evenness (Fig. 3C) was lower in Summer than Torpor (Tukey’s HSD adj *P* = 0.022). Shannon’s diversity did not differ among groups (Fig. 3D, ANOVA adj *P* = 0.868). Content microbiota results (Additional file 1: Fig. S5) were the same as for the mucosa microbiota with respect to the number of observed OTUs and Shannon’s diversity index; however, phylogenetic diversity and phylogenetic evenness differed. Phylogenetic diversity only displayed a trend of differences among groups (ANOVA adj *P* = 0.069). Phylogenetic evenness was lower in Summer compared to Torpor or IBA (Tukey’s HSD both adj *P* < 0.001), and lower in Spring compared to Torpor (adj *P* = 0.042).

**Fig. 4:**
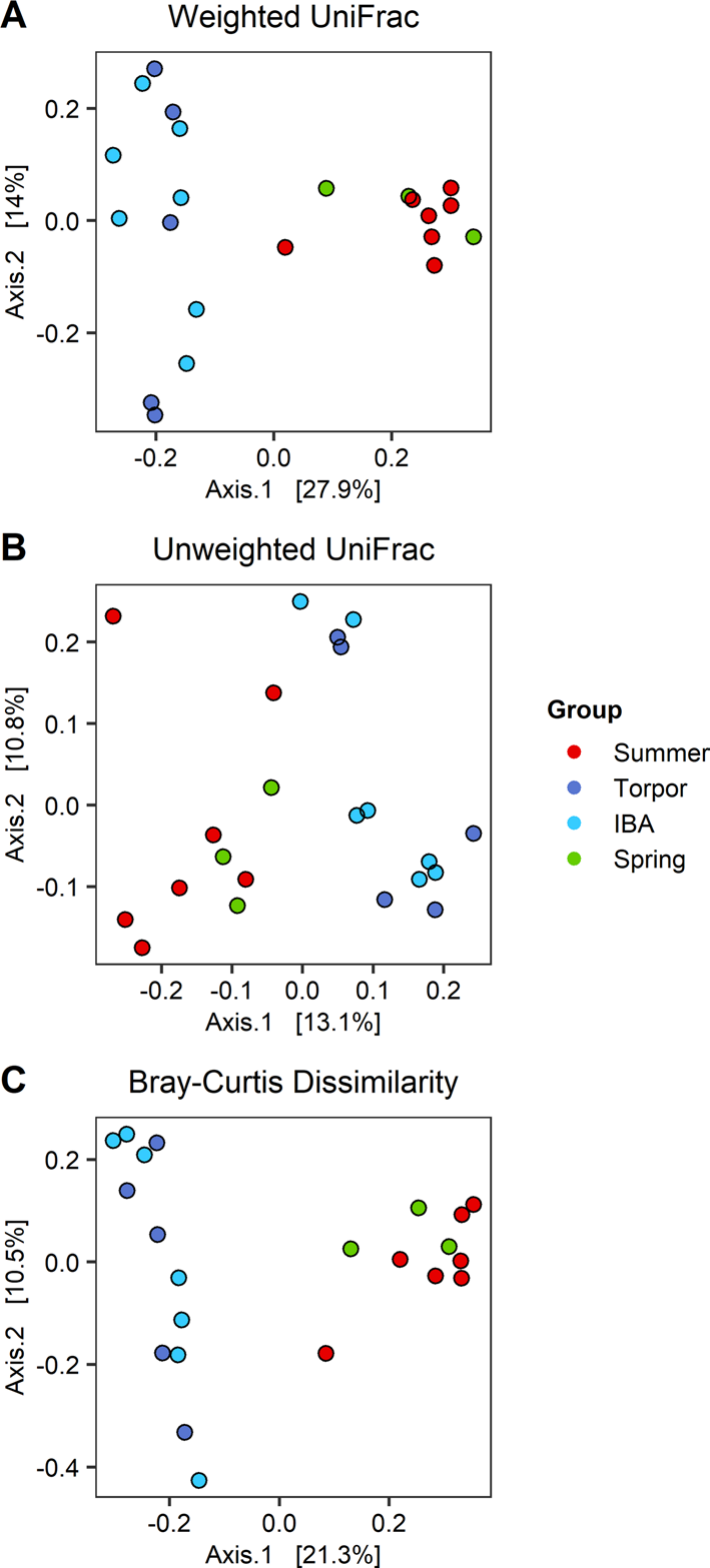
Beta diversity of mucosa microbiotas from captive groups. **A** Weighted UniFrac, **B** unweighted UniFrac, and **C** Bray–Curtis dissimilarity are displayed on PCoA ordinations. Groups are depicted with different colors

Beta diversity variance across all captive mucosa microbiotas (Fig. 4) did not differ in weighted and unweighted UniFrac and in Bray–Curtis dissimilarity (all betadisper *P* ≥ 0.822). However, the centroids of active groups (Summer and Spring) were different from those of winter groups (Torpor and IBA) in weighted UniFrac (all PERMANOVA adj *P* < 0.005) and in Bray–Curtis dissimilarity (all adj *P* < 0.027). The unweighted UniFrac centroids differed between Summer and winter groups (Torpor adj *P* = 0.008, IBA adj *P* = 0.003), and between Spring and IBA (adj *P* = 0.033). There were no differences within active and winter groups (both adj *P* for weighted UniFrac ≥ 0.143, unweighted UniFrac ≥ 0.635, and Bray–Curtis dissimilarity ≥ 0.094). Content microbiota beta diversity results (Additional file 1: Fig. S6) were similar to those of mucosa microbiotas, except that the unweighted UniFrac centroids of Torpor and Spring were different (adj *P* = 0.038). Lastly, we attempted to identify OTUs that drove these differences in beta diversity by using SIMPER but detected no significant OTUs (all adj *P* ≥ 0.075).

## Discussion

In this study, we sought to determine if the bacterial cecal microbiotas of TLGS were impacted by captive versus wild environments. We compared summer microbiotas between TLGS born in captivity that consumed a chow diet with wild-caught TLGS that consumed a natural, wild diet. We found that Wild microbiotas had greater richness and phylogenetic diversity and decreased variance in beta diversity compared to Captive microbiotas. Important taxa that contributed to differences in beta diversity and were core OTUs were primarily classified to the families *Lachnospiraceae* and *Ruminococcaceae*. We also described the microbiotas of captive TLGS across the year (summer, winter, and spring) using Illumina-based sequencing as most previous rodent hibernation microbiota studies to date have used either 454 pyrosequencing [56–59] or clone libraries [60].

Animals born and raised in the wild experience several environmental features that can affect their gut microbiota composition and therefore influence the host-microbe symbiosis. The composition and availability of food is arguably the most important environmental influence as these can affect early microbiota development and host-microbe relationships throughout life [12, 13, 16, 17]. Although commercial animal chow provides adequate macro- and micro-nutrients for generalized rodents, precise recapitulation of the wild diet is difficult, especially for omnivores like the TLGS. Foraging in the wild also provides opportunities for animals to ingest microbes that contribute to their gut microbiota. This variable is largely missing in captive diets, which can have significant effects on microbiota diversity and the host-microbe symbiosis [48].

Another potential variable is host age. Studying animals in captivity is useful because it provides precise age information that is often unavailable for wild animals. In our study, we were unable to assign exact ages to the Wild group; however, based on their body weights at capture (Additional file 2: Table S1), we predicted that they were likely adults of at least 13 months of age. Therefore, the Wild squirrels were all older than the Captive squirrels. Previous TLGS microbiota studies have found that there are few differences between the summer microbiotas of wild-caught mothers and the summer microbiotas of their captive pups that were born and raised in captivity with known ages [56, 57]. Therefore, we posit that age differences between our Wild and Captive TLGS would not significantly bias our results, but future studies should account for this variable.

Additional limitations of our study include the use of small sample sizes and relative abundance data. Due to our small sample sizes, we only have sufficient power to detect very large effect sizes that were often larger than our observed effect sizes (Additional file 2: Tables S4 and S5) [65, 66]. Although the power of our study is limited, our work still provides crucial information to help future studies calculate the number of samples needed to have sufficient statistical power. Future studies can reference our observed effect sizes to determine sample sizes for both wild and captive ground squirrels, whereas such data was not previously available. Additionally, our use of relative abundance data may be misleading as changes in the abundance of one taxa will influence the relative abundances of all other taxa in that sample. This is especially important in hibernating ground squirrels because absolute bacterial abundance dramatically decreases in hibernation compared to active seasons [59].

Overall, we found that differences in the alpha diversity of content and mucosa microbiotas between the Captive and Wild groups were attributed to the Wild group’s larger number of OTUs with low relative abundances. The Wild group had significantly more observed OTUs (Fig. 1A) and higher phylogenetic diversity (Fig. 1B) compared to Captive. These additional Wild OTUs were from numerous different taxa rather than from a single or closely related group of taxa as there was no difference in the average phylogenetic distance among all species pairs (Fig. 1C). Most of the Wild OTUs also had low relative abundances, demonstrated by the lack of difference in Shannon’s index (Fig. 1D).

Beta diversity of content and mucosa microbiotas between Wild and Captive groups were different with respect to OTU identities, relative abundances, and phylogenetic relatedness (Fig. 2). Wild content microbiotas also had less variation than Captive content microbiotas; in mucosa microbiotas, this was true only in unweighted UniFrac. Because diet is a known driver of the microbiota, we expected the Wild group to have higher variation compared to the Captive group, as reported in a previous study that examined three species of hibernating bats [45]. However, we found the opposite to be true. This was consistent with another study that found captive rodent microbiotas had more variation than wild rodent microbiotas [53]. One possible explanation is that the wild squirrels were captured from the same area on the same day, so their diets may have been very similar. Additionally, the small sample size of the Wild group may underestimate beta diversity variation.

Although Captive and Wild microbiotas had different alpha and beta diversities, we detected the same phyla in both groups, with no significant differences in relative abundances (Table 1); however, we did identify trends in the *Proteobacteria* and *Cyanobacteria* in content microbiotas. The *Proteobacteria* displayed a trend of higher relative abundance in Captive than in Wild microbiotas, which contrasted with results in other wild rodent studies where the *Proteobacteria* have higher relative abundances in wild compared to captive animals [6, 42, 67]. The top *Proteobacteria* OTUs in Captive microbiotas included members of the family *Desulfovibrionaceae*, such as the genera *Desulfovibrio* and *Parasutterella*. *Desulfovibrio* is a sulfate-reducer known to participate in mucin glycan degradation [68] and increases in relative abundance during fasting [60] and hibernation [56, 59]. *Parasutterella* isolates from rodent and human guts are asaccharolytic and predicted to rely on amino acids to survive [69]; they also induce changes in bile acid metabolism and aromatic amino acid catabolism when introduced in mice [69]. The *Cyanobacteria* had higher relative abundance in Captive than in Wild microbiotas. All TLGS *Cyanobacteria* OTUs were classified to the class *Melainabacteria* and the order *Gastranaerophilales*. *Melainabacteria*, a proposed sister phylum to Cyanobacteria, is reported to not be autotrophic [70–72], has been detected in diverse environments ranging from the human gut to groundwater [70], and is predicted to produce formate and ethanol in the gut [70].

We identified the families *Lachnospiraceae* and *Ruminococcaceae* as the predominant classifications of core OTUs in both Captive and Wild microbiotas (Additional file 2: Tables S5 and S6) and of OTUs that explained Bray–Curtis dissimilarity (Table 2 and Additional file 2: Table S4). Members of both families are known to degrade diverse plant polysaccharides [73, 74]. There were more core OTUs in Wild compared to Captive microbiotas, which is not surprising given that Wild microbiotas had more observed OTUs; however, this could also be due to its small sample size (n = 4). Just under half of the Captive core OTUs were also Wild core OTUs, demonstrating that there is reasonable overlap between the two groups despite their different alpha and beta diversities. Bray–Curtis dissimilarity between Captive and Wild microbiotas was explained by several *Lachnospiraceae* OTUs that had higher relative abundances in Wild than in Captive microbiotas, which is consistent with what has been observed in wild and captive mice [31, 39]. These taxa likely help the host metabolize plant material, as members of the *Lachnospiraceae* are known for degrading diverse polysaccharides and are positively correlated with acetate, butyrate, and propionate in TLGS [56].

It is important to note that most rodent hibernation microbiota studies have used either 454 pyrosequencing [56–59] or clone libraries [60]. However, advances in sequencing technology have resulted in the vast majority of microbiota studies using Illumina-based sequencing. Because different sequencing technologies are known to introduce various biases [61–63], we considered if seasonal changes in the microbiotas of captive TLGS across the year (summer, winter, and spring) were consistent between Illumina sequencing and pyrosequencing. Overall, our findings from Illumina-based sequencing were generally consistent with those based on 454 pyrosequencing. We identified more phyla than in previous studies, but only found changes in the relative sequence abundances of the dominant phyla, and not in those with lower relative abundances [56, 57]. This suggests that trends in taxa with high relative abundances are conserved regardless of sequencing technology, but there may be differences in taxa with low relative abundances. These differences may also be due to different primers used in one of the previous studies [57]. We also confirmed previous findings that a small number of core OTUs persist in the TLGS cecal microbiota throughout the year despite seasonal shifts in the host’s diet [57]. Finally, our results with respect to alpha and beta diversity in winter (Torpor and IBA) and active groups (Summer and Spring) were similar, although we note that there were some minor differences in the gut microbiotas of active group squirrels. Taken together, these data confirm previous findings on the TLGS gut microbiota and address concerns that may arise due to biases inherent to the two sequencing technologies.

## Conclusions

This is the first study to compare the microbiotas of captive and wild rodent hibernators. We demonstrated that Wild TLGS microbiotas have increased richness and phylogenetic diversity, and decreased variation in beta diversity compared to Captive TLGS microbiotas. In both Captive and Wild microbiotas, important taxa that are core OTUs or significantly contribute to beta diversity are predominantly in the families *Lachnospiraceae* and *Ruminococcaceae*. Given the significant differences between wild and captive TLGS microbiotas, it is important that future research determines how long it takes for wild microbiotas to change in captivity and what those changes entail. We also reported seasonal differences in captive TLGS microbiotas throughout the annual cycle by using Illumina-based 16S rRNA gene sequencing. Results about alpha diversity, beta diversity, and taxa with high relative abundances were consistent with past conclusions based on 454 pyrosequencing, but some differences emerged when comparing Summer and Spring seasons and in the analysis of low abundance taxa. Because next-generation sequencing continues to advance quickly, continued methodological comparisons should be conducted to evaluate any biases introduced by new methods. Taken together, our results help improve reproducibility and experimental design of future hibernation microbiota studies.

## Methods

### Animals

**Fig. 5:**
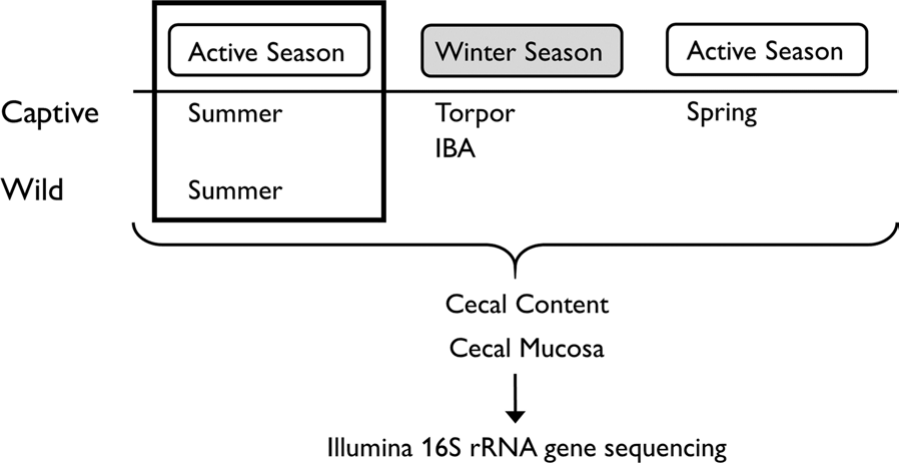
Experimental design. Cecal content and mucosa were collected from summer captive and wild TLGS, as well as captive TLGS across the year. The black box indicates the two summer groups that were used in the comparison of Captive and Wild microbiotas. For the description of captive TLGS across seasons, four groups were used: Summer, Torpor, IBA, and Spring. Summer and Spring groups represent active seasons, while Torpor and IBA groups represent the winter season

All procedures were approved by the University of Wisconsin-Madison Institutional Animal Care and Use Committee under protocols V001229 and V005481. Two groups of healthy TLGS were used in our study: a captive and a wild group (Fig. 5). The captive group consisted of 26 animals (17 females and 9 males) that were born in captivity to seven pregnant, wild-caught females in Madison, WI. The pregnant females were housed individually at 22 °C with a 12:12 h light–dark cycle, provided water and rat chow (Harlan Teklad no. 7001, Indianapolis, IN, USA) ad libitum, and supplemented with apples, strawberries, and sunflower seeds weekly. Pups were born in May 2016 and remained with their mothers for five weeks before separation into new cages that housed two pups per cage. After two weeks, pups were transferred into individual cages. Water and chow were provided ad libitum for two weeks. Then chow was restricted to 12 g/day to prevent excessive weight gain and sunflower seeds (~ 1 g) were provided weekly. The wild squirrel group consisted of four adult TLGS (two males and two females) captured in July 2017 in Madison, WI.

### Sampling Regimen

The 26 captive TLGS were randomly assigned to one of four groups (Fig. 5): Summer (n = 8), Torpor (n = 6), IBA (n = 8), or Spring (n = 4). Captive Summer squirrels were sampled in August 2016. The remaining captive animals were transferred to a 4 °C room in September/October 2016 for hibernation. The room was held in constant darkness except for a daily, brief period of dim light (~ 5 min) to check activity states using the sawdust method [75]. This involved placing very small pieces of paper on top of a torpid squirrel and examining if the paper had been disturbed, which would indicate that a squirrel had entered an IBA and then reentered torpor since the previous check. Once squirrels began using torpor, food and water were removed. Winter squirrels were sampled in January–February (after ~ 3.5–4.5 months in the cold room). Torpor squirrels were sampled during torpor (T_b_ < 10 °C). For IBA squirrels, torpid animals were brought to a lit 22 °C room for three hours to induce an arousal and sampled when T_b_ > 34 °C. Spring squirrels were removed from the cold room in January 2017 and transferred to a warm room with food and water ad libitum for two weeks before sampling in February 2017. Samples were collected from wild summer squirrels on the same day they were captured in July 2017. Metadata for all squirrels are listed in Additional file 2: Table S1.

### Sample collection

Four anesthesia/euthanasia methods were used due to equipment availability and sample collection requirements for a separate project. Captive Summer and Spring groups were euthanized via exposure to isoflurane for 5–15 min followed by decapitation. Torpor squirrels were euthanized by decapitation or cervical dislocation. IBA squirrels were anesthetized via exposure to isoflurane or CO_2_ for 5–15 min followed by decapitation (Additional file 2: Table S1). Wild summer squirrels were euthanized with pentobarbital followed by decapitation. To determine whether anesthesia method had a significant impact on the gut microbiota, IBA squirrels that received isoflurane or CO_2_ were compared as this was the only experimental group where multiple anesthesia methods were used (Additional file 2: Table S2). We did not find evidence that the microbiotas of isoflurane- or CO_2_-administered animals were different. The methods [76–78] and results for this analysis are shown in Additional file 2: Table S2.

T_b_ was measured immediately by inserting a clean thermal probe into the body cavity. Cecal contents were collected by removing intact ceca and gently scraping the content into a sterile tube. To remove any remaining content, cecal tissue was rinsed with sterile phosphate-buffered saline (PBS) and any remaining liquid was gently scraped off. Cecal mucosae were collected using a razor blade to gently scrape off the mucosa. To assess the captive diet microbiota, three chow samples were collected. A representative wild diet was not collected as TLGS are omnivorous and consume a diverse diet that includes various plants, insects, and small birds [79]. All samples were placed on dry ice and stored at − 80 °C. We collected a total of 62 samples: 30 cecal content samples (26 captive, 4 wild), 29 cecal mucosa samples (25 captive, 4 wild), and three chow samples.

### DNA extraction

Total genomic DNA was extracted from each sample using a phenol:chloroform extraction protocol [80] with the following modification: all aqueous phase washes used 25:24:1 phenol:chloroform:isoamyl alcohol instead of phenol:chloroform for a total of four washes. Four controls were processed with the cecal samples. Two sample collection controls were aliquots from the sterile PBS used to rinse cecal tissue. These were uncovered and exposed to air for the same amount of time needed to collect the cecal samples. Two extraction method controls contained sterile water. DNA was quantified with the Qubit Fluorometer (Invitrogen, San Diego, CA, USA) and stored at − 80 °C.

### DNA amplification and sequencing

We used universal bacterial primers flanking the V4 region of the 16S rRNA gene (515F: GTGCCAGCMGCCGCGGTAA, 806R: GGACTACHVGGGTWTCTAAT) [81, 82]. The primers also contained adapters (forward: AATGATACGGCGACCACCGAGATCTACAC, reverse: CAAGCAGAAGACGGCATACGAGAT) compatible with Illumina sequencing technology (Illumina, San Diego, CA, USA) and unique barcodes for multiplexing (forward: eight unique eight bp barcodes, reverse: 12 unique eight bp barcodes). Each reaction contained 50 ng DNA, 0.4 μM forward primer, 0.4 μM reverse primer, 12.5 μL 2X HotStart ReadyMix (KAPA Biosystems, Wilmington, MA, USA), and water to a final volume of 25 μL. Polymerase chain reaction (PCR) was performed using a Bio-Rad S1000 thermocycler (Bio-Rad Laboratories, Hercules, CA, USA). Cycling conditions began with initial denaturation at 95 °C for 3 min, followed by 25 cycles of 95 °C for 30 s, 55 °C for 30 s, and 72 °C for 30 s, and a final extension at 72 °C for 5 min. Four controls consisting of sterile water were processed alongside sample DNA to ensure there was no contamination during PCR. PCR products were purified using gel extraction from a 1.0% low-melt agarose gel (National Diagnostics, Atlanta, GA, USA) with a ZR-96 Zymoclean DNA Recovery Kit (Zymo Research, Irvine, CA, USA) and DNA was quantified using a Qubit Fluorometer. 60 samples were successfully amplified, and two samples failed to amplify (both chow samples). Samples were equimolarly pooled with 10% PhiX control DNA and sequenced on an Illumina MiSeq using a MiSeq 2 × 250 v2 kit.

### Microbiota sequence clean-up

Sequences were demultiplexed on the Illumina MiSeq and processed using mothur v1.44.3 [83] and SILVA release 132 [84–87]. Fastq files were submitted to NCBI’s Short Read Archive and are publicly available under accession number PRJNA742778. Mothur logfiles and output files are found at https://github.com/ednachiang/CaptiveWild/tree/master/mothur_output.

We followed the mothur standard operating procedure accessed on March 19, 2021 with the following modifications: we used *screen.seqs* maxlength = 300 and *chimera.uchime*. Briefly, the procedure involved combining paired reads, removing poor quality reads, aligning reads to SILVA release 132 [84–87], and removing undesirable reads (chimeras, Archaea, Eukaryota, chloroplasts, mitochondria, and unknown). Sequences were grouped into 97% operational taxonomic units (OTUs). Samples were normalized to 11,013 sequences using a modified total sum scaling method that optimized sample coverage. First, the proportion of each OTU relative to total reads was calculated separately for each sample. To ensure that all samples had sufficient coverage, a read cutoff was selected based on the sample with the lowest number of sequences that resulted in all normalized samples having Good’s coverage ≥ 95% [64]. The OTU proportions were then multiplied by the sequence cutoff and rounded to the nearest integer. Three samples were removed due to insufficient sequencing (Good’s coverage < 95%): one chow, one Torpor mucosa, and one summer Wild content sample. All eight control samples were also discarded due to low number of sequences (mean = 32; range 3–117). To create a phylogenetic tree, a representative sequence from each OTU was first selected using *get.oturep* and the most abundant sequence. Representative sequences were renamed to match their assigned OTU and a phylip-formatted distance matrix was calculated. This distance matrix was used to calculate a phylogenetic tree using *clearcut* and the tree was classified using *classify.tree*.

### Statistical analysis in R

Outputs from mothur were imported into R version 3.4.3 [88] using the phyloseq [89] and phytools [90] packages. All visualizations were created using the ggplot2 [91], grid [88], and gridExtra [92] packages. The following packages were also used for statistical analyses: ape [93], dplyr [94], dunn.test [95], effsize [96], equivalence [97], picante [98], pwr [99], stats [88], tidyr [100], and vegan [101].

We used different subsets of samples to perform three analyses. To compare cecal content and mucosa microbiotas, only squirrels with both sample types were included due to the use of paired tests, and samples within each experimental group were analyzed separately. To compare Captive and Wild microbiotas, only summer Captive and summer Wild squirrels were considered, and content and mucosa samples were analyzed separately. Lastly, to compare captive microbiotas across the year (summer, winter, and spring), we analyzed captive groups (Summer, Torpor, IBA, and Spring) separately for content and mucosa samples. In all three comparisons, OTUs found in less than three samples were removed. Four outlier samples were also removed: content and mucosa samples from one IBA and one Spring individual (Additional file 2: Table S3). These were identified as samples with: (1) alpha diversity that was more than 1.5 times the interquartile range above the upper quartile or below the lower quartile for its experimental group, and (2) large beta diversity distances that, in an ordination, clearly clustered away from all other samples in the same experimental group. Additional information about the four outlier samples is found in Additional file 2: Table S3. All code to replicate statistical analyses and generate figures is available at https://github.com/ednachiang/CaptiveWild.

### Alpha diversity analysis

We evaluated alpha diversity (within-sample diversity) using the number of observed OTUs, Faith’s phylogenetic diversity, phylogenetic evenness, and Shannon’s weighted diversity index [102]. The number of observed OTUs was calculated using the phyloseq package [89]. Faith’s phylogenetic diversity was computed by taking the sum of all branches in a tree using the picante package (*pd*) [98]. Because phylogenetic diversity is positively correlated with richness, we included a metric unbiased by richness: mean pairwise distance (MPD), which measures phylogenetic evenness [103]. MPD represents the average phylogenetic distance between all species pairs. Positive values indicate more phylogenetic evenness while negative values indicate less phylogenetic evenness. MPD was calculated by comparing phylogenetic diversity to a null model over 999 iterations (*cophenetic*, stats package [88]; *ses.mpd*, picante package [98]). Lastly, Shannon’s weighted diversity index was calculated in mothur [83]. We tested the normality of each alpha diversity metric using quantile–quantile plots and the Shapiro test (*qqnorm, qqline*, *shapiro.test*, stats package [88]) before selecting a statistical test.

To compare content and mucosa microbiotas, paired t-tests were used as all metrics had normal distributions. Similarly, t-tests were used to compare Captive and Wild microbiotas. To compare captive microbiotas across seasons, content samples were analyzed using analysis of variance (ANOVA) (*aov,* stats package [88]) followed by pairwise Tukey’s Honest Significant Difference tests (*TukeyHSD*, stats package [88]). Mucosa MPD and Shannon’s diversity index were also analyzed this way. Mucosa number of observed OTUs and phylogenetic diversity were not normally distributed and were therefore analyzed using Kruskal–Wallis (*kruskal.test*, stats package [88]) followed by Dunn’s Test (*dunn.test*, dunn.test package [95]). All *p*-values were adjusted for false discovery rate using the Benjamini–Hochberg procedure (*p.adjust*, stats package [88]). Results were considered significant if adj *P* < 0.05 except for Dunn’s test where adj *P* ≤ 0.025 was significant.

### Beta diversity analysis

Beta diversity (between-sample diversity) was examined using weighted UniFrac, unweighted UniFrac, and Bray–Curtis dissimilarity. All three metrics were calculated using the phyloseq package (*distance*) [89] and visualized using principal coordinate analysis (PCoA) ordinations (*ordinate*, phyloseq package [89]). To test whether there were significant differences in variance/dispersion, homogeneity of groups dispersions tests (*betadisper*, vegan package [101]) were used. Next, we tested if beta diversity centroids differed among experimental groups using permutational multivariate analysis of variance [104] (PERMANOVA; *adonis*, vegan package [101]). All *p*-values were corrected for false discovery rate using the Benjamini–Hochberg procedure.

To identify which OTUs significantly contributed to differences in Bray–Curtis dissimilarity, the similarity percentages test (SIMPER; *simper*, vegan package [101]) was used along with the Kruskal–Wallis test with false discovery rate correction using the Benjamini–Hochberg procedure. Only OTUs that accounted for ≥ 1% of the differences in beta diversity with adj *P* < 0.05 were considered.

### Phylum-level analysis

Phylum-level relative abundances were calculated using the phyloseq [89] and dplyr [94] packages and phyla with total relative abundances < 1% were removed. Prior to selecting a statistical test, we evaluated normality using quantile–quantile plots and Shapiro tests. For the Captive and Wild comparison, phyla with normal distributions were analyzed using t-tests, whereas those with non-normal distributions were analyzed using Wilcoxon Rank Sum tests (*wilcox.test*, stats package [88]). To compare captive microbiotas across seasons, phyla with normal distributions were analyzed using ANOVA followed by Tukey’s HSD and those with non-normal distributions were analyzed using Kruskal–Wallis followed by Dunn’s test. All *p*-values were corrected for false discovery rate using the Benjamini–Hochberg procedure and results were considered significant if adj *P* < 0.05, except for Dunn’s test where adj *P* ≤ 0.025 was significant.

### Identification of core OTUs

Core OTUs were defined as OTUs that were present in every sample within a group and were identified using the phyloseq package [89]. Core OTUs shared among groups were identified by taking the intersect of the core OTUs in two or more groups.

### Calculation of effect size and power

Effect size and power were calculated for alpha diversity comparisons between paired content and mucosa samples (Additional file 2: Table S4), and between summer Captive and Wild samples (Additional file 2: Table S5). They were also calculated for comparisons of phyla relative abundances between summer wild and captive samples (Additional file 2: Table S5). Effect size (Cohen’s d) was calculated with *cohen.d* (effsize package [96]), and power was calculated with *pwr.t2n.test* (pwr package [99]).

## Supplementary Information

**Additional file 1.** **Figure S1.** Alpha diversity of content and mucosa microbiotas within each experimental group. Violin plots display the number of observed OTUs (first column), Faith’s phylogenetic diversity (second column), phylogenetic evenness (mean pairwise distance (MPD); third column), and Shannon’s diversity (fourth column) for each experimental group: (A – D) Summer Wild, (E – H) Summer Captive, (I – L) Torpor, (M – P) IBA, (Q – T) Spring. There were no significant comparisons (all t-test adj P ≥ 0.889). **Figure S2.** Beta diversity of content and mucosa microbiotas within each experimental group. Weighted UniFrac (first column), unweighted UniFrac (second column), and Bray-Curtis dissimilarity (third column) are displayed on PCoA ordinations for each group: (A – C) Summer Wild, (D – F) Summer Captive, (G – I) Torpor, (J – L) IBA, (M – O) Spring. **Figure S3.** Alpha diversity between summer Captive and Wild content microbiotas. Violin plots display four alpha diversity metrics: (A) the number of observed OTUs, (B) Faith’s phylogenetic diversity, (C) phylogenetic evenness (MPD), and (D) Shannon’s diversity. An asterisk indicates a significant difference (t-test adj P < 0.05) and “ns” indicates no significant difference (adj P > 0.05). **Figure S4.** Beta diversity of summer Captive and Wild content microbiotas. (A) Weighted UniFrac, (B) unweighted UniFrac, and (C) Bray-Curtis dissimilarity are displayed on principal coordinate analysis PCoA ordinations. Groups are depicted with different colors. **Figure S5.** Alpha diversity comparison among content microbiotas across captive groups. Violin plots display four alpha diversity metrics: (A) the number of observed OTUs, (B) Faith’s phylogenetic diversity, (C) phylogenetic evenness (MPD), and (D) Shannon’s diversity. Groups that share a letter are not significantly different (Tukey’s HSD adj P > 0.05), whereas groups that share no letters are significant different (adj P < 0.05). Metrics with no significant comparisons between groups are indicated with “ns." **Figure S6.** Beta diversity of content microbiotas in captive groups. (A) Weighted UniFrac, (B) unweighted UniFrac, and (C) Bray-Curtis dissimilarity are displayed on principal coordinate analysis PCoA ordinations. Groups are depicted with different colors.

**Additional file 2.** **Table S1.** Squirrel metadata. **Table S2.** Gut microbiota comparison of squirrels that received isoflurane or CO_2_ for anesthesia. To determine whether anesthesia had a significant impact on the gut microbiota, we examined IBA squirrels as this was the only group in which more than one anesthesia method was used. We compared IBA animals that received isoflurane or CO_2_ using the robust two one-sided test (RTOST) of equivalence [84, 102–104]. RTOST is a nonparametric test whose null and alternative hypotheses are switched compared to that of a traditional t-test: the null hypothesis is that two means are not equivalent and the alternative hypothesis is that two means are equivalent. Using OTU tables, each sample from a squirrel that received CO_2_ was compared to the mean of all samples from squirrels that received isoflurane (Table S1). P-values were adjusted for false discovery rate using the Benjamini-Hochberg procedure. All comparisons were significant; therefore, we rejected our null hypothesis that the means (or microbiotas of isoflurane- and CO_2_ - administered squirrels) are not equivalent. **Table S3.** Metadata and alpha diversity metrics for the four outlier samples. **Table S4.** Effect size and power for alpha diversity comparisons between paired content and mucosa samples in each experimental group. Effect sizes were calculated in R using cohen.d (effsize package [83]), and power was calculated using pwr.t2n.test (pwr package [86]). **Table S5.** Effect size and power for Wild vs. Captive group comparisons of alpha diversity and phyla relative abundances. Effect sizes were calculated in R using cohen.d (effsize package [83]), and power was calculated using pwr.t2n.test (pwr package [86]). **Table S6.** PERMANOVA results from beta diversity comparison of content and mucosa beta diversity in each experimental group. Three beta diversity metrics (weighted UniFrac, unweighted UniFrac, and Bray-Curtis dissimilarity) and two variables (squirrel ID and sample type) were tested. **Table S7.** Phyla relative abundances in summer Captive and Wild content microbiotas. Phyla have total relative abundances > 1% and are displayed from overall highest relative abundance to lowest relative abundance. Relative abundances are displayed as mean ± standard error and adjusted p-values are from t-tests or Wilcoxon Rank Sum tests, depending on data normality. **Table S8.** OTUs that significantly contribute to Bray-Curtis dissimilarity between Captive and Wild content microbiotas. OTUs were identified with SIMPER and statistically tested with Kruskal-Wallis tests. P-values were corrected for false discovery rate using the Benjamini-Hochberg procedure. Only OTUs that accounted for ≥ 1% of the differences in beta diversity and had adj P < 0.05 were considered significant. **Table S9.** Core OTUs in summer Captive and Wild mucosa microbiotas. X's in a Captive or Wild column indicate that the OTU is a core OTU in that group, while empty cells indicate that the OTU is not a core OTU in the group. **Table S10.** Core OTUs in summer Captive and Wild content microbiotas. X's in a Captive or Wild column indicate that the OTU is a core OTU in that group, while empty cells indicate that the OTU is not a core OTU in the group. **Table S11.** Phyla relative abundances in content microbiotas of captive groups. Phyla have total relative abundance >1% and are displayed from overall highest relative abundance to lowest relative abundance. Relative abundances are displayed as mean ± standard error and adjusted p-values are from ANOVA and Tukey's HSD tests, or Kruskal-Wallis and Dunn's tests, depending on data normality. **Table S12.** Core OTUs in mucosa microbiotas across captive groups. X's in a captive group column indicate that the OTU is a core OTU in that group, while empty cells indicate that the OTU is not a core OTU in the group. **Table S13.** Core OTUs in content microbiotas across captive groups. X's in a captive group column indicate that the OTU is a core OTU in that group, while empty cells indicate that the OTU is not a core OTU in the group.

## Data Availability

All FAST Q files were submitted to the NCBI’s Short Read Archive and are publicly available under bioproject number PRJNA742778 (https://www.ncbi.nlm.nih.gov/bioproject/PRJNA742778). Mothur logfiles and output files, as well as all R code used to perform statistical analyses and generate figures, are publicly available at https://github.com/ednachiang/CaptiveWild.

## Abbreviations

ANOVA: Analysis of variance
IBA: Interbout arousal
MPD: Mean pairwise distance
OTU: Operational taxonomic unit
PBS: Phosphate-buffered saline
PCoA: Principal coordinate analysis
PCR: Polymerase chain reaction
PERMANOVA: Permutational multivariate analysis of variance
SIMPER: Similarity percentages test
T_b_: Body temperature
TLGS: 13-Lined ground squirrel
