# Supplementary material for: Characterization of captive and wild 13-lined ground squirrel cecal microbiotas using Illumina-based sequencing
Source: Anim Microbiome. 2022 Jan 3;4:1. doi: 10.1186/s42523-021-00154-9 (PMC8722175; doi:10.1186/s42523-021-00154-9)
Supplement: Supplementary file 1 — Additional file 1. Figure S1. Alpha diversity of content and mucosa microbiotas within each experimental group. Violin plots display the number of observed OTUs (first column), Faith’s phylogenetic diversity (second column), phylogenetic evenness (mean pairwise distance (MPD); third column), and Shannon’s diversity (fourth column) for each experimental group: (A – D) Summer Wild, (E – H) Summer Captive, (I – L) Torpor, (M – P) IBA, (Q – T) Spring. There were no significant comparisons (all t-test adj P ≥ 0.889). Figure S2. Beta diversity of content and mucosa microbiotas within each experimental group. Weighted UniFrac (first column), unweighted UniFrac (second column), and Bray-Curtis dissimilarity (third column) are displayed on PCoA ordinations for each group: (A – C) Summer Wild, (D – F) Summer Captive, (G – I) Torpor, (J – L) IBA, (M – O) Spring. Figure S3. Alpha diversity between summer Captive and Wild content microbiotas. Violin plots display four alpha diversity metrics: (A) the number of observed OTUs, (B) Faith’s phylogenetic diversity, (C) phylogenetic evenness (MPD), and (D) Shannon’s diversity. An asterisk indicates a significant difference (t-test adj P < 0.05) and “ns” indicates no significant difference (adj P > 0.05). Figure S4. Beta diversity of summer Captive and Wild content microbiotas. (A) Weighted UniFrac, (B) unweighted UniFrac, and (C) Bray-Curtis dissimilarity are displayed on principal coordinate analysis PCoA ordinations. Groups are depicted with different colors. Figure S5. Alpha diversity comparison among content microbiotas across captive groups. Violin plots display four alpha diversity metrics: (A) the number of observed OTUs, (B) Faith’s phylogenetic diversity, (C) phylogenetic evenness (MPD), and (D) Shannon’s diversity. Groups that share a letter are not significantly different (Tukey’s HSD adj P > 0.05), whereas groups that share no letters are significant different (adj P < 0.05). Metrics with no significant comparisons between [file 42523_2021_154_MOESM1_ESM.docx]

**Additional file 1**

**
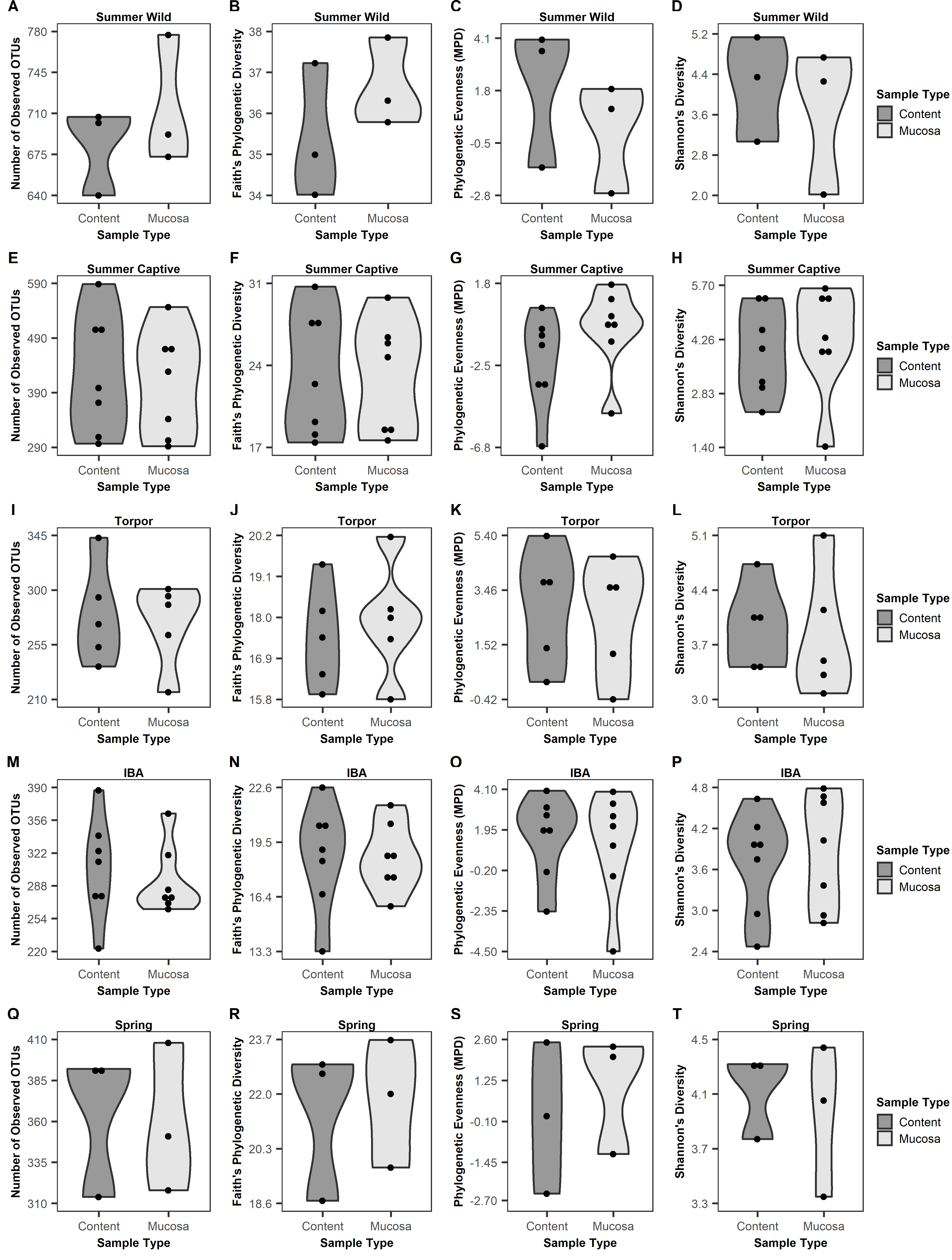
**

**Figure S1. Alpha diversity of content and mucosa microbiotas within each experimental group.** Violin plots display the number of observed OTUs (first column), Faith’s phylogenetic diversity (second column), phylogenetic evenness (mean pairwise distance (MPD); third column), and Shannon’s diversity (fourth column) for each experimental group: (A – D) Summer Wild, (E – H) Summer Captive, (I – L) Torpor, (M – P) IBA, (Q – T) Spring. There were no significant comparisons (all t-test adj P ≥ 0.889).

**
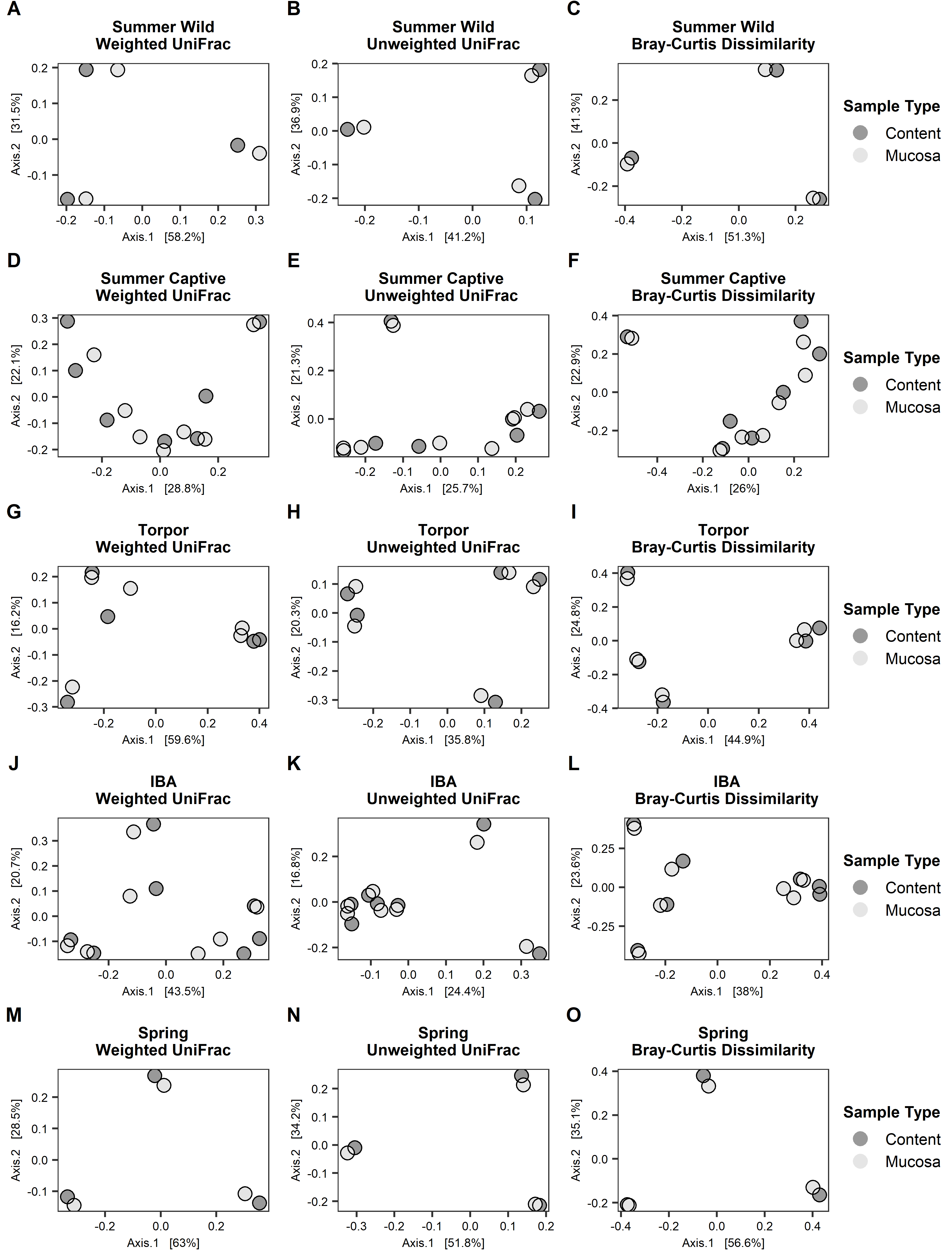
**

**Figure S2. Beta diversity of content and mucosa microbiotas within each experimental group*.*** Weighted UniFrac (first column), unweighted UniFrac (second column), and Bray-Curtis dissimilarity (third column) are displayed on PCoA ordinations for each group: (A – C) Summer Wild, (D – F) Summer Captive, (G – I) Torpor, (J – L) IBA, (M – O) Spring.

**
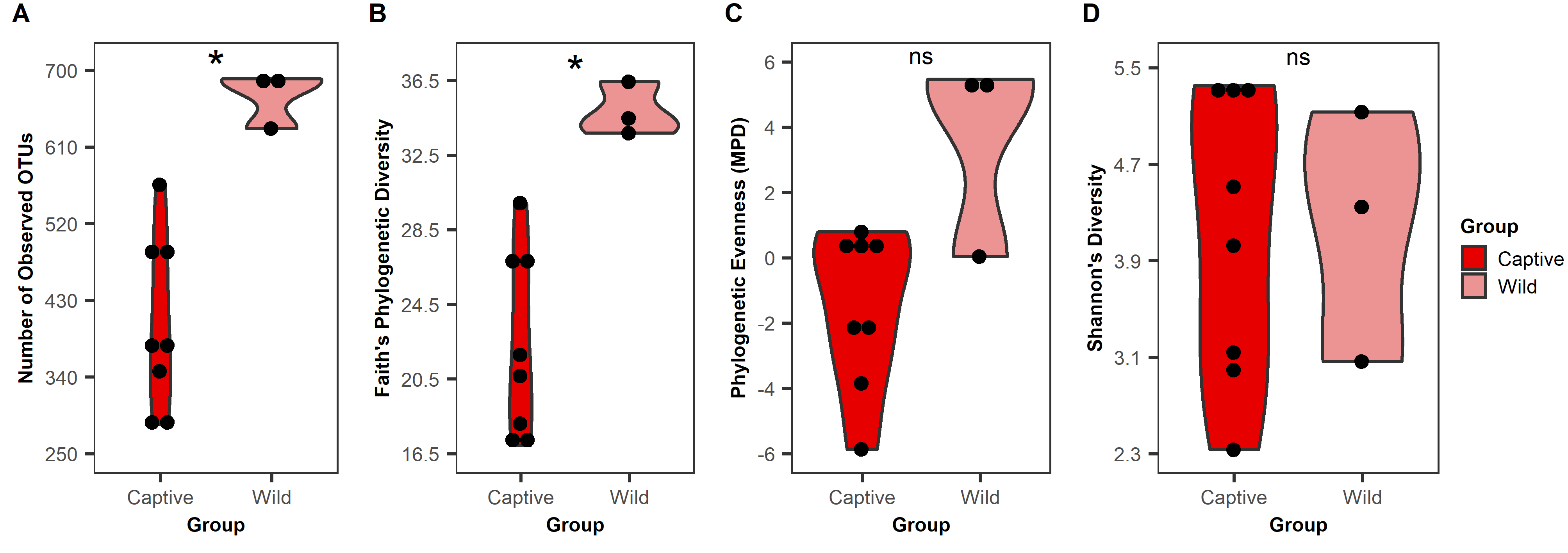
**

**Figure S3. Alpha diversity between summer Captive and Wild content microbiotas.** Violin plots display four alpha diversity metrics: (A) the number of observed OTUs, (B) Faith’s phylogenetic diversity, (C) phylogenetic evenness (MPD), and (D) Shannon’s diversity. An asterisk indicates a significant difference (t-test adj P < 0.05) and “ns” indicates no significant difference (adj P > 0.05).

**
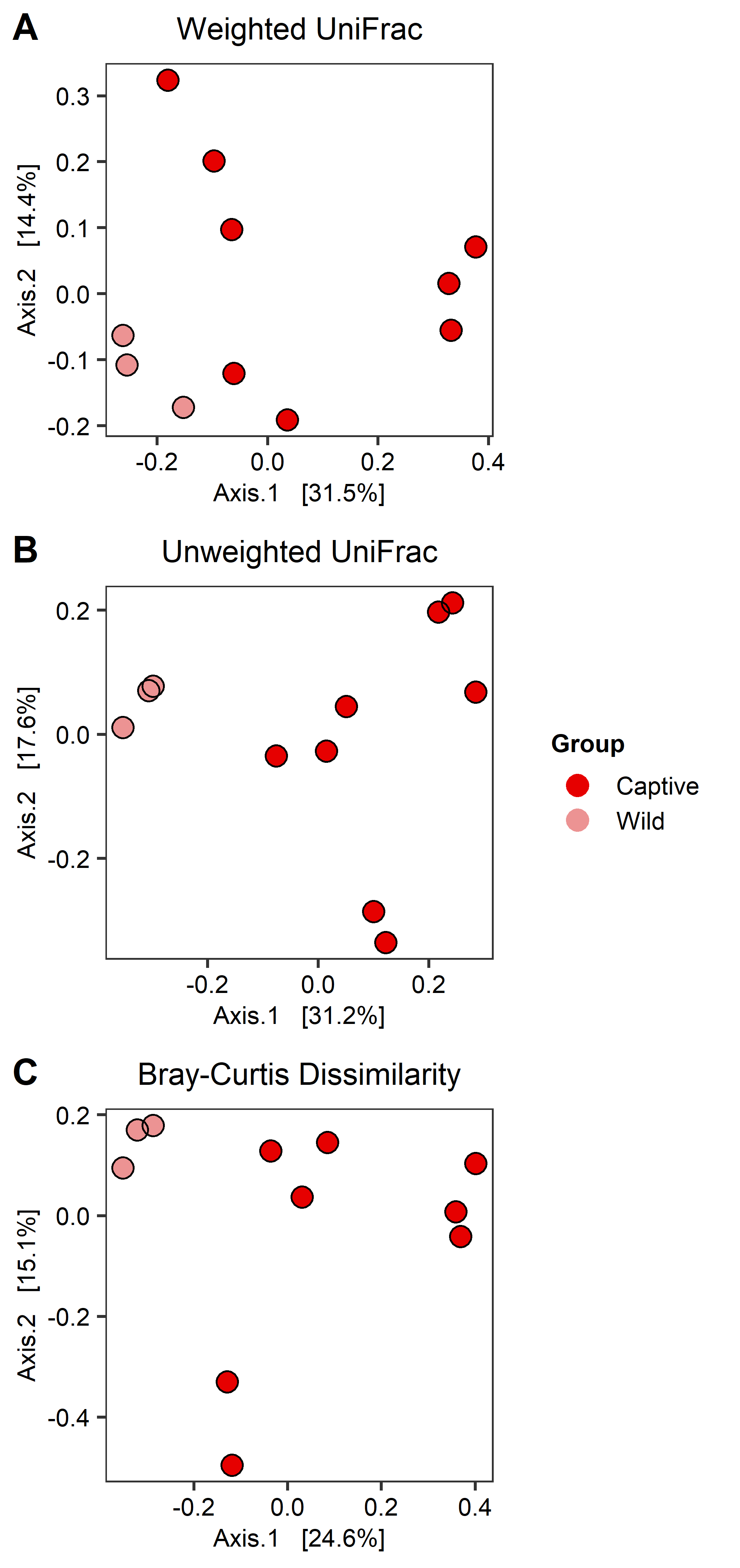
**

**Figure S4. Beta diversity of summer Captive and Wild content microbiotas.** (A) Weighted UniFrac, (B) unweighted UniFrac, and (C) Bray-Curtis dissimilarity are displayed on principal coordinate analysis PCoA ordinations. Groups are depicted with different colors.


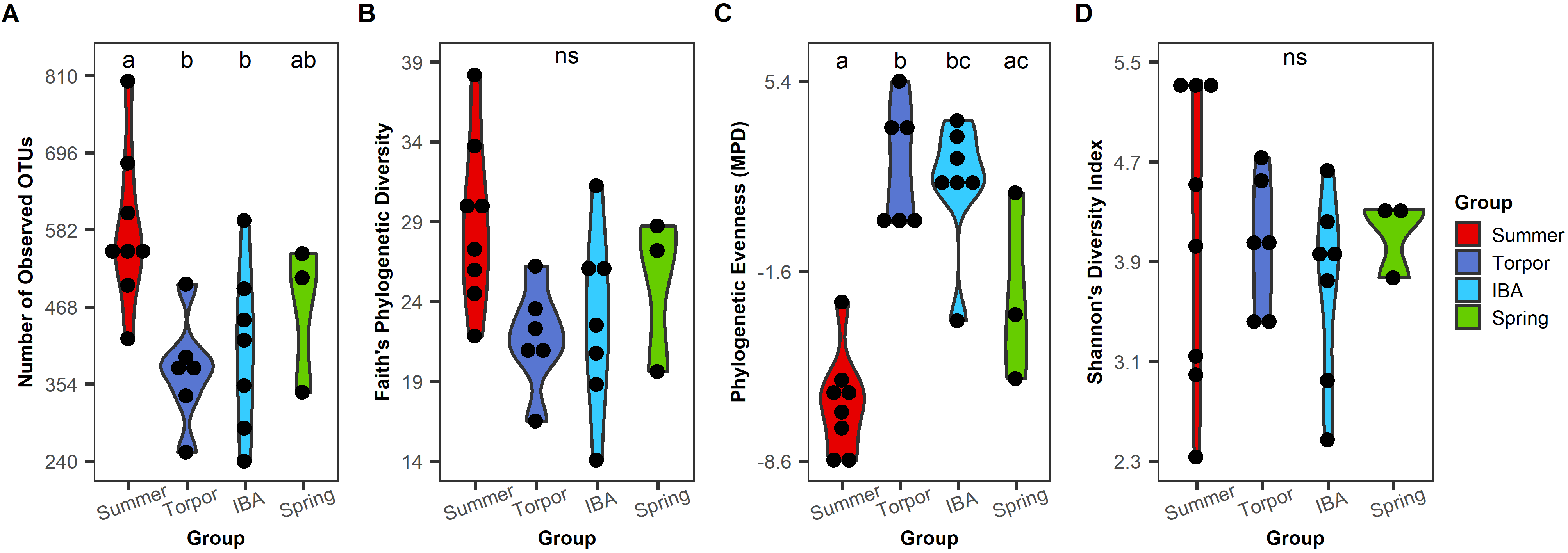


**Figure S5. Alpha diversity comparison among content microbiotas across captive groups.** Violin plots display four alpha diversity metrics: (A) the number of observed OTUs, (B) Faith’s phylogenetic diversity, (C) phylogenetic evenness (MPD), and (D) Shannon’s diversity. Groups that share a letter are not significantly different (Tukey’s HSD adj P > 0.05), whereas groups that share no letters are significant different (adj P < 0.05). Metrics with no significant comparisons among groups are indicated with “ns.”

**
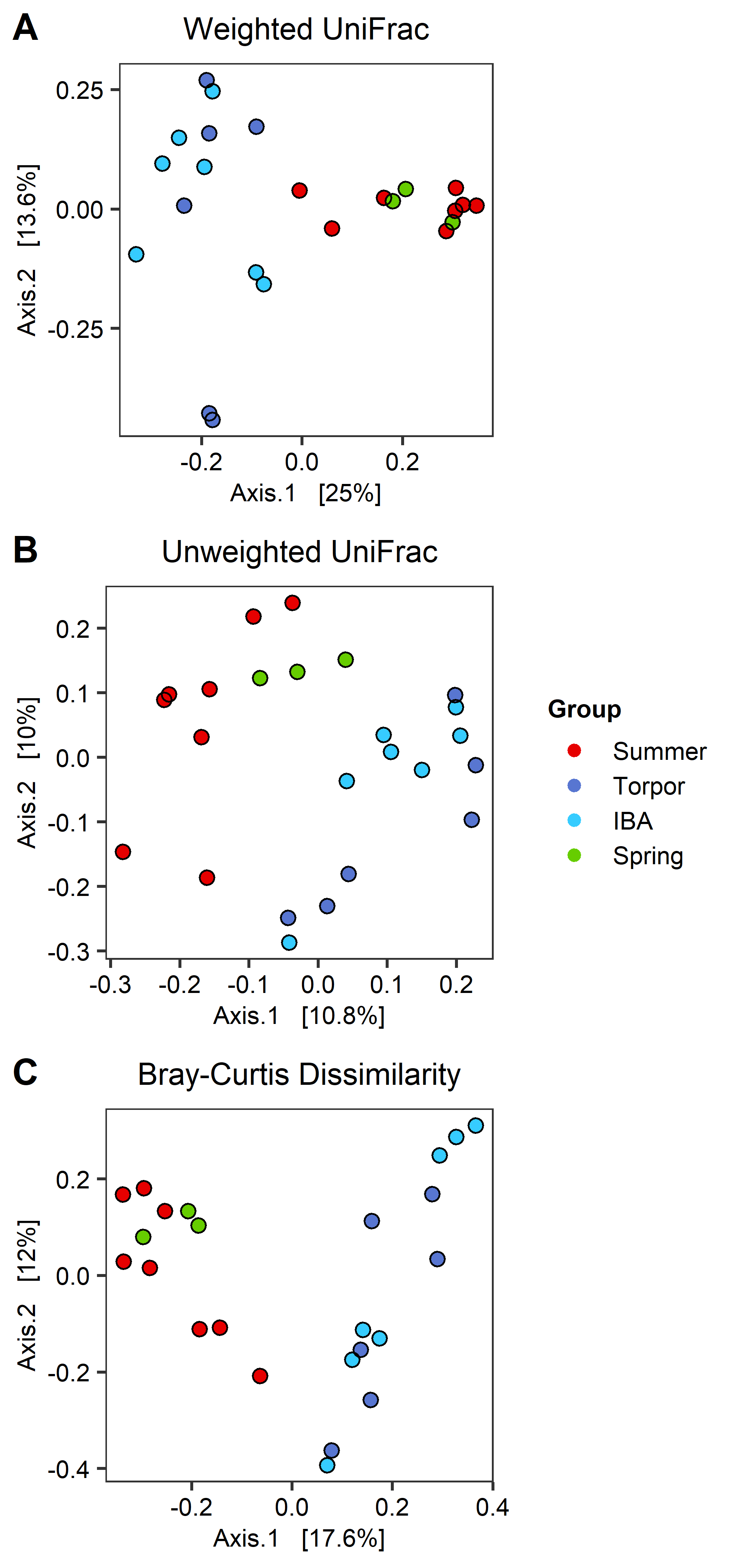
**

**Figure S6. Beta diversity of content microbiotas in captive groups.** (A) Weighted UniFrac, (B) unweighted UniFrac, and (C) Bray-Curtis dissimilarity are displayed on principal coordinate analysis PCoA ordinations. Groups are depicted with different colors.

**Table S1. Squirrel metadata.**

**Table S2. Gut microbiota comparison of squirrels that received isoflurane or CO2 for anesthesia**. To determine whether anesthesia had a significant impact on the gut microbiota, we examined IBA squirrels as this was the only group in which more than one anesthesia method was used. We compared IBA animals that received isoflurane or CO_2_ using the robust two one-sided test (RTOST) of equivalence [84, 102-104]. RTOST is a nonparametric test whose null and alternative hypotheses are switched compared to that of a traditional t-test: the null hypothesis is that two means are not equivalent and the alternative hypothesis is that two means are equivalent. Using OTU tables, each sample from a squirrel that received CO_2_ was compared to the mean of all samples from squirrels that received isoflurane (Table S1). P-values were adjusted for false discovery rate using the Benjamini-Hochberg procedure. All comparisons were significant; therefore, we rejected our null hypothesis that the means (or microbiotas of isoflurane- and CO_2_-administered squirrels) are not equivalent.

**Table S3. Metadata and alpha diversity metrics for the four outlier samples.**

**Table S4. Effect size and power for alpha diversity comparisons between paired content and mucosa samples in each experimental group.** Effect sizes were calculated in R using *cohen.d* (effsize package [83]), and power was calculated using *pwr.t2n.test* (pwr package [86]).

**Table S5. Effect size and power for Wild vs. Captive group comparisons of alpha diversity and phyla relative abundances.** Effect sizes were calculated in R using *cohen.d* (effsize package [83]), and power was calculated using *pwr.t2n.test* (pwr package [86]).

**Table S6. PERMANOVA results from beta diversity comparison of content and mucosa beta diversity in each experimental group.** Three beta diversity metrics (weighted UniFrac, unweighted UniFrac, and Bray-Curtis dissimilarity) and two variables (squirrel ID and sample type) were tested.

**Table S7. Phyla relative abundances in summer Captive and Wild content microbiotas.** Phyla have total relative abundances > 1% and are displayed from overall highest relative abundance to lowest relative abundance. Relative abundances are displayed as mean ± standard error and adjusted p-values are from t-tests or Wilcoxon Rank Sum tests, depending on data normality.

**Table S8. OTUs that significantly contribute to Bray-Curtis dissimilarity between Captive and Wild content microbiotas.** OTUs were identified with SIMPER and statistically tested with Kruskal-Wallis tests. P-values were corrected for false discovery rate using the Benjamini-Hochberg procedure. Only OTUs that accounted for ≥ 1% of the differences in beta diversity and had adj P < 0.05 were considered significant.

**Table S9. Core OTUs in summer Captive and Wild mucosa microbiotas.** X's in a Captive or Wild column indicate that the OTU is a core OTU in that group, while empty cells indicate that the OTU is not a core OTU in the group.

**Table S10. Core OTUs in summer Captive and Wild content microbiotas.** X's in a Captive or Wild column indicate that the OTU is a core OTU in that group, while empty cells indicate that the OTU is not a core OTU in the group.

**Table S11. Phyla relative abundances in content microbiotas of captive groups**. Phyla have total relative abundance >1% and are displayed from overall highest relative abundance to lowest relative abundance. Relative abundances are displayed as mean ± standard error and adjusted p-values are from ANOVA and Tukey's HSD tests, or Kruskal-Wallis and Dunn's tests, depending on data normality.

**Table S12. Core OTUs in mucosa microbiotas across captive groups.** X's in a captive group column indicate that the OTU is a core OTU in that group, while empty cells indicate that the OTU is not a core OTU in the group.

**Table S13. Core OTUs in content microbiotas across captive groups.** X's in a captive group column indicate that the OTU is a core OTU in that group, while empty cells indicate that the OTU is not a core OTU in the group.
